# First person – Akanksha Onkar

**DOI:** 10.1242/dmm.050505

**Published:** 2023-10-19

## Abstract

First Person is a series of interviews with the first authors of a selection of papers published in Disease Models & Mechanisms, helping researchers promote themselves alongside their papers. Akanksha Onkar is first author on ‘ Increase in brain glycogen levels ameliorates Huntington's disease phenotype and rescues neurodegeneration in *Drosophila*’, published in DMM. Akanksha conducted the research described in this article while a Ph.D. scholar in Dr S. Ganesh's lab at Indian Institute of Technology Kanpur, Kanpur, India. She is now a postdoctoral researcher in the lab of Dr Adrian Erlebacher at University of California San Francisco, investigating the moonlighting roles of carbohydrates in normal physiology and disease states.


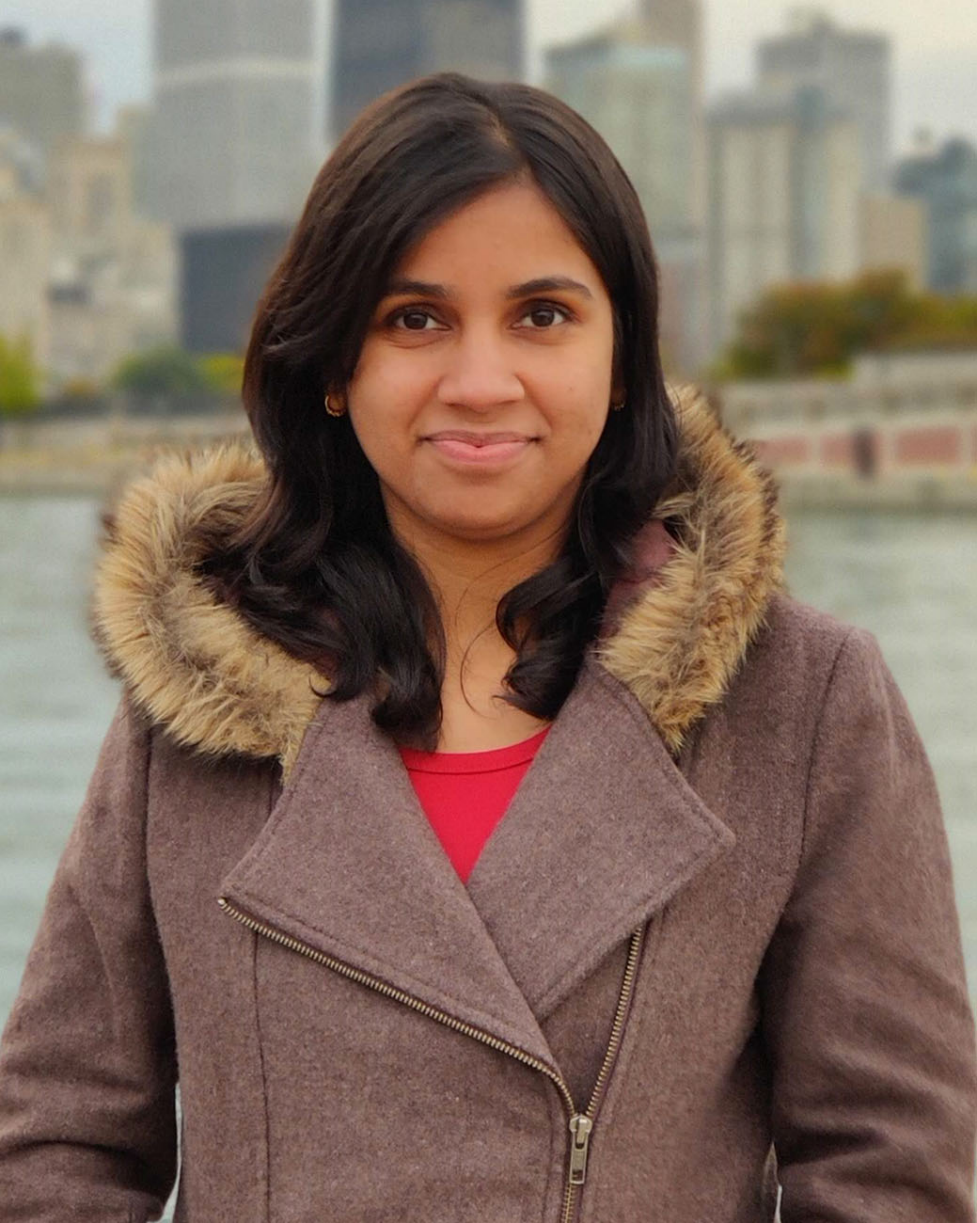


**Akanksha Onkar**

Intriguingly, the brain of an aging individual or of someone with a neurodegenerative disorder has been shown to accumulate enormous amounts of glycogen.

**How would you explain the main findings of your paper to non-scientific family and friends?**

Glycogen, a glucose polymer, though present in all major organs as an energy reservoir, is scarce in the brain. This phenomenon of lower glycogen levels contrasts with the brain's high energy requirements and need for a continuous glucose supply. Intriguingly, the brain of an aging individual or of someone with a neurodegenerative disorder has been shown to accumulate enormous amounts of glycogen. In this study, we have tried to solve this paradox of glycogen content differences in the aging/diseased brain using the fly model of the neurodegenerative disorder Huntington's disease (HD). HD is characterized by mutant huntingtin protein aggregates, resulting in neuronal death and life-threatening symptoms in animal models and patients. Indeed, in the current work, we observed that ectopic expression of mutant huntingtin protein in the brain resulted in its aggregate formation and reduced lifespan in the flies. Interestingly, we also observed increased brain glycogen levels and increased activity of the enzyme responsible for glycogen synthesis, i.e. glycogen synthase (GS), which paralleled the HD disease progression. To understand why the HD brain would choose to make glycogen, we ectopically expressed or knocked down GS in the HD brain of fruit flies. We observed that, on the one hand, strengthening GS by overexpression increased lifespan in the HD flies and improved behavioral deficits associated with HD. Knockdown of GS, on the other hand, led to a more aggressive disease phenotype in the HD flies by increasing the levels of huntingtin aggregates. We also identified a GS-mediated induction of the cells' waste clearance pathway autophagy and the improvement of defects in lysosomes as the key to glycogen/GS-dependent neuroprotection in the HD brain. Our current findings confirm that a degenerating HD brain tends to accumulate glycogen as a defense strategy to combat the disease and to increase the chances of the organisms' survival and, thus, could be the reason that glycogen accumulation is familiar to many neurodegenerative diseases.

**What are the potential implications of these results for your field of research?**

We have investigated the role of glycogen in HD in the *Drosophila* model of the disease, which – so far – is one of the rare pieces of evidence showing the neuroprotective influence of the metabolite in an *in-vivo* system. Our study bestows a novel role to glycogen in neurodegeneration, an effect beyond glycogens' known function as only acting as a glucose reservoir. Thus, the study can help investigators design therapies for HD by targeting the glycogen synthesis pathway in animal models and humans. The study also opens avenues to investigate the effect of glycogen and its synthesis machinery in the context of aging and other neurodegenerative disorders, where, though glycogen accumulation has been reported, the reason behind its presence remains elusive. Therefore, given the protective influence of glycogen observed in our current work and the presence of glycogen in the aging/neurodegenerative brain identified in previous studies, we suspect the glycogen synthesis pathway could be a generic target for either or both scenarios.

**What are the main advantages and drawbacks of the experimental system you have used as it relates to the disease you are investigating?**

The experimental model in our work is *Drosophila melanogaster*, which has pros and cons. The UAS-*Gal4* system in *Drosophila* allowed us to modulate the brain-specific expression of GS, thus permitting the investigation of molecular mechanisms and interactions in a spatially controlled fashion. HD modeling in the fruit fly mimics most of the observed in other animal models of the disease and even human patients, like retinal degeneration, motor deficits and lifespan shortening. Another riveting advantage is the shorter lifespan of the fly, leading to our discovery of brain glycogen-mediated effects on fitness and reproductive traits in HD, thus, highlighting the involvement of brain glycogen in the neuroendocrine-gonadal axis, a feature that is difficult to study if not controlled spatially. Despite the innumerable benefits of using the fruit fly for the current work, our HD model has yet to capture the entire spectrum of the disease, and requires validation in animal models and humans.

[…], it was astounding to notice the decrease in the mutant huntingtin aggregation in the HD brain upon the ectopic expression of GS, and the aggregates became bigger upon GS knockdown.

**Figure:**
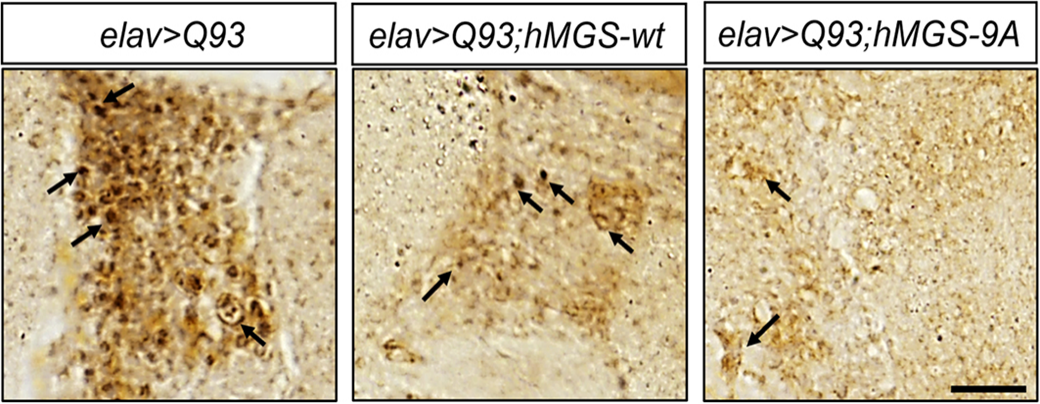
**Bright-field images showing aggregates of mutant huntingtin protein in the brain of 1-week old flies.** *Drosophila* brains expressed mutant huntingtin alone (*elav>Q93*), mutant huntingtin together with the wild-type form of glycogen synthase (*elav>Q93;hMGS-wt*) or mutant huntingtin together with the constitutively active form of glycogen synthase (*elav>Q93;hMGS-9A*). Arrows indicate huntingtin aggregate puncta. Scale bar: 50 µm.

**What has surprised you the most while conducting your research?**

While conducting this work, two things specifically amazed me. First was the enormous glycogen accumulation we observed in the brain upon inducing the expression of the mutant huntingtin protein, unlike when the wild-type huntingtin protein was overexpressed. These results beautifully depicted how proteotoxic stress could trigger glycogen synthesis in the brain. Second, it was astounding to notice the decrease in the mutant huntingtin aggregation in the HD brain upon the ectopic expression of GS, and the aggregates became bigger upon GS knockdown. These findings ensured that the brain tended to make glycogen as a survival mechanism and highlighted the unusual role of glycogen in maintaining proteo-stasis in the brain under stress.

**Describe what you think is the most significant challenge impacting your research at this time and how will this be addressed over the next 10 years?**

While studying HD and glycogen, we faced challenges initially with the choice of which model to study. We looked into the mouse model repository and were surprised by the need for better models for the HD. The HD mouse models available commercially are global knockouts, in which studying the spatial effect of GS, and differentiating between primary and secondary outcomes of our gene manipulations was challenging. Further, there is a lack of commercially available mouse models in which GS expression can be perturbed or modified throughout or in a spatiotemporal fashion. At this point, the HD model fruit fly came to our rescue, and we were able to study the unique role of GS in HD pathology. Surprisingly, most neurodegenerative diseases suffer from a similar lack of model systems, specifically mice models, which are necessary to cover the broad spectrum of the disease, as some cannot be replicated in invertebrate model systems. Thus, there is a definite need to introduce more robust neurodegenerative disease models to understand the conditions better and design translational therapies. Where systems like Cre-lox recombination can help understand the spatial and temporal function of neurodegenerative disease-associated genes, the advent of methods like CRISPR-Cas9 can help attain these outcomes efficiently.

**What changes do you think could improve the professional lives of scientists?**

With the advent and inclusivity of techniques like single-cell sequencing and other computational knowledge-based methods in scientific studies, most researchers often need help to learn the ever-emerging programming languages. Firstly, introducing a mandatory biology-specific programming course in the researchers' curriculum is quintessential, along with constant communication and collaboration with experts in the field. Another aspect of improving the professional lives of scientists is realizing the importance of developing soft skills, like communication, presentation and the art of scientific articulation, so that researchers can explain even complicated work to the general public and help make a world where science is for all.

**What's next for you?**

While working on glycogen during my Ph.D., I realized my love for carbohydrates and the unique functions they could perform in various contexts. Thus, as a postdoctoral fellow, I have embarked on a new journey of understanding the role of carbohydrates in maintaining immune tolerance towards the fetus during pregnancy. By combining my knowledge of sugars with the concepts of feto-maternal tolerance, I wish to contribute to a better understanding of both entities for scientists to work on.
